# Adjunctive betamethasone treatment of hypoxaemic adults hospitalised with *Mycoplasma pneumoniae* community-acquired pneumonia: an open-label, multicentre, randomised, controlled trial

**DOI:** 10.1016/j.lanepe.2026.101610

**Published:** 2026-04-19

**Authors:** Karl Hagman, Magnus Hedenstierna, Elina Andersson Norlén, Karin Biasoletto, Maria Eklund Josephson, Carl-Johan Fraenkel, Elin Hedman, Johan Ljungberg, Oskar Ljungquist, Viktor Månsson, David Nygren, Milena de Oliveira e Costa, Cecilia Rydén, Jørgen Skov Jensen, Göran Stenlund, Jonas Tverring, Lisa Wasserstrom, Anna C. Nilsson, Johan Ursing

**Affiliations:** a Department of Infectious Diseases, Sahlgrenska University Hospital, Gothenburg, Region Västra Götaland, Sweden; b Department of Infectious Diseases, Institute of Biomedicine, Sahlgrenska Academy at University of Gothenburg, Gothenburg, Sweden; c Department of Infectious Diseases, Danderyd Hospital, Stockholm, Sweden; d Department of Infectious Diseases, Östersund Hospital, Östersund, Sweden; e Department of Clinical Microbiology, Umeå University, Umeå, Sweden; f Department of Infectious Diseases, Skåne University Hospital, Malmö, Sweden; g Department of Infectious Diseases, Skåne University Hospital, Lund, Sweden; h Division of Infection Medicine, Department of Clinical Sciences, Lund University, Sweden; i Department of Infectious Diseases, Hospital of Halland, Halmstad, Sweden; j Department of Infectious Diseases, Helsingborg Hospital, Helsingborg, Sweden; k Clinical Infection Medicine, Department of Translational Medicine, Lund University, Malmö, Sweden; l Department of Medicine, Capio S:t Göran Hospital, Stockholm, Sweden; m Department of Bacteria, Parasites, and Fungi, Statens Serum Institut, Denmark; n Department of Infectious Diseases, Malarhospital, Eskilstuna, Sweden; o Clinical Microbiology, Department of Translational Medicine, Faculty of Medicine, Lund University, Malmö, Sweden; p Clinical Microbiology, Infection Control and Prevention, Skåne University Hospital, Lund, Sweden; q Department of Clinical Sciences, Danderyd Hospital, Karolinska Institutet, Stockholm, Sweden

**Keywords:** Atypical pneumonia, Corticosteroids, Oxygen, Antibiotics, Doxycycline

## Abstract

**Background:**

Adjunctive corticosteroid therapy appears to be beneficial for adults with severe community-acquired pneumonia (CAP), but data on *Mycoplasma pneumoniae* CAP are limited. This study aimed to evaluate if adjunctive betamethasone reduced time to resolution of hypoxaemia in adults hospitalised with *M. pneumoniae* CAP.

**Methods:**

An open-label, multicentre, randomised, controlled trial was conducted at eight Swedish hospitals. Adults admitted with *M. pneumoniae* CAP and hypoxaemia (SpO_2_ <93% and respiratory rate >20 breaths/min) were eligible. Exclusion criteria included asthma and diabetes mellitus. Participants were randomised (1:1) to either standard care without corticosteroids or adjunctive oral betamethasone (3 mg daily on days 1–2, and 2 mg daily on days 3–5). Antibiotic treatment was standardised to 200 mg doxycycline once daily for 10 days. The primary outcome was time to resolution of hypoxaemia in an intention-to-treat analysis. Safety was assessed in participants receiving at least one betamethasone dose. The study was registered with the EU Clinical Trials Register (EudraCT 2016-002585-32) and is completed.

**Findings:**

Between March 1, 2018, and November 14, 2024, 70 participants were enrolled: 36 in the betamethasone group and 34 in the control group. The median age was 42 years (IQR 28–49) including 40 (57%) male and 30 (43%) female participants. All participants achieved resolution of hypoxaemia except two who withdrew consent. Time to resolution of hypoxaemia was shorter in the betamethasone group (HR 1.82 [95% CI 1.10–3.02], p = 0.020) compared to the standard of care group, with an estimated median of 2.3 (95% CI 1.8–2.7) and 3.6 (95% CI 1.9–5.3) days, respectively. Adverse events were similar between study groups. Severe adverse events were rare, and none were attributed to betamethasone treatment.

**Interpretation:**

Adjunctive betamethasone was well tolerated and significantly shortened the duration of hypoxaemia in adults hospitalised with *M. pneumoniae* CAP in this open-label trial.

**Funding:**

The Swedish Society of Medicine.

**Research in context**

**Evidence before this study**

We searched PubMed from inception to September 11, 2025, using the terms (“*Mycoplasma pneumoniae*”) AND (corticosteroid), without language restrictions. In paediatric populations, a recent meta-analysis reported potential benefit with high-dose, compared to low-dose, adjunctive corticosteroid therapy in children with severe *M. pneumoniae* community-acquired pneumonia (CAP). However, evidence in adults is limited to two retrospective cohort studies, both reporting no or limited benefits of corticosteroid treatment.

**Added value of this study**

This is the first pathogen-specific, randomised controlled trial evaluating adjunctive corticosteroid therapy in hypoxaemic adults (n = 70) hospitalised with *M. pneumoniae* CAP. Participants randomised to a five-day course of oral betamethasone (total dose 12 mg) had significantly shorter time to resolution of hypoxaemia (HR 1.8 [95% CI 1.1–3.0], p = 0.020) compared to those randomised to standard care without corticosteroids. The estimated median duration of hypoxaemia was 2.3 days (95% CI 1.8–2.7) and 3.6 days (95% CI 1.9–5.3), respectively. Time to hospital discharge was also shorter (HR 2.1 [95% CI 1.2–3.4], p = 0.007) for patients in the betamethasone group, and adverse events were comparable between groups.

**Implications of all the available evidence**

Evidence suggests that corticosteroid therapy with moderate-dose betamethasone in addition to standard antibiotics may offer clinically meaningful benefits for hypoxaemic adults with *M. pneumoniae* CAP, including shorter duration of hypoxaemia. Corticosteroids should thus be considered as part of the treatment strategy in hypoxaemic adults. Further research is warranted to assess efficacy in adults with milder disease.

## Introduction

*Mycoplasma pneumoniae* is one of the most common bacterial causes of community-acquired pneumonia (CAP) accounting for approximately 2% of hospitalisations due to pneumonia in adults.^1^ This corresponds to an incidence rate of 8.5 cases per 100,000 person-years (95% Confidence Interval [CI] 7.9–9.2).^1^ Adults with *M. pneumoniae* CAP are typically younger, have fewer comorbidities, and lower mortality rates compared to those with other causes of CAP.^2^ Historically, *M. pneumoniae* infections occur with epidemic peaks approximately every four years.^3^ A marked decline in reported cases during the SARS-CoV-2 pandemic was followed by a global resurgence with unprecedented high numbers of *M. pneumoniae* in late 2023.^4^

In a meta-analysis, adjunctive corticosteroid therapy was shown to reduce mortality in patients with severe all-cause CAP.^5^ The relevance to *M. pneumoniae* CAP of these findings remain uncertain since only few *M. pneumoniae* cases were included in the analysed studies, and the mortality associated with this pathogen is low. High-dose corticosteroid therapy was reported to show potential benefit in paediatric populations with severe *M. pneumoniae* CAP, but data regarding adults are limited.^6^ No beneficial effects of corticosteroid use on mortality or length of stay were reported in a retrospective cohort of adults (N = 2228) hospitalised with *M. pneumoniae* CAP.^7^ Similarly, corticosteroid use was not associated with time to resolution of hypoxaemia or length of stay in a cohort of 388 hypoxaemic patients, although a shorter duration of fever was noted.^8^ High-dose adjunctive corticosteroids were associated with a significantly increased risk of adverse events, raising concerns about their use in this disease with generally favourable outcome.^7^

This randomised, controlled trial aimed to investigate the if moderate-dose betamethasone as an adjunctive to antibiotic therapy reduced time to resolution of hypoxaemia in adults hospitalised with *M. pneumoniae* CAP.

## Methods

### Study design

This open label, randomised, controlled, multicentre clinical trial was conducted at eight Swedish hospitals. The Regional Ethical Review Board in Stockholm (2017/114-31) and the Swedish Medical Products Agency (5.1-2017-73584) approved the protocol which is available in the Supplementary Appendix. The trial was registered with the European Union Drug Regulating Authorities Clinical Trials Database (EudraCT number 2016-002585-32) and is completed.

### Participants

Patients with *M. pneumoniae* CAP admitted to a study hospital were considered for study entry. Patients were identified by notification from the respective hospitals’ microbiological laboratories and by referral from clinicians. The participating microbiological laboratories are accredited according to ISO 15189.

Inclusion criteria were: (1) age ≥18 years, (2) *M. pneumoniae* infection defined as positive *M. pneumoniae* PCR on a sample taken from upper or lower respiratory tract, (3) infiltrates on medical imaging consistent with pneumonia, (4) pneumonia not acquired at hospital, (5) admitted to a study hospital, (6) hypoxaemia defined as having a peripheral oxygen saturation (SpO_2_) below 93% (measured by pulse oximetry), and a breathing rate of >20 per minute without supplemental oxygen treatment, (7) a negative pregnancy test taken before inclusion and use of an acceptable effective method of contraception until treatment discontinuation if the participant was a woman of childbearing potential, (8) written informed consent after meeting with a study physician, and ability and willingness to complete follow up.

Exclusion criteria were: (1) significant growth of alternative lower airway pathogen such as *Streptococcus pneumoniae* or *Haemophilus influenzae* in sputum, (2) known current gastric ulcer, (3) pregnancy or breast-feeding, (4) diabetes mellitus, (5) chronic obstructive pulmonary disease or asthma, (6) ongoing corticosteroid treatment, (7) hypersensitivity to any ingredient in the study drugs, (8) inability to give informed consent, or (9) significantly compromised immunity. Compromised immunity included but was not limited to treatment with major immunosuppressive agents including high dose corticosteroids, anti-TNF agents, calcineurin inhibitors, mTOR inhibitors, lymphocyte depleting biological agents, or chemotherapeutic anti neoplastic agents. Moreover, patients with advanced HIV/AIDS, severe immunodeficiency such as hypogammaglobulinemia, decompensated liver cirrhosis, and bone marrow transplant during the last year were not eligible for inclusion.

### Randomisation

Participants were randomised by study personnel to either a betamethasone group or a control group using www.sealedenvelope.com (Sealed Envelope Ltd., UK). Randomisation was done using computer generated, random permuted blocks of two to six participants to ensure that the groups were balanced periodically with 1:1 allocation ratio, without any stratification. Treatment was not blinded.

### Procedures

The antibiotic treatment was standardised to doxycycline (ATC J01AA02) 200 mg once daily for all participants upon trial entry, for a total of 10 days of effective treatment.

Participants assigned to the treatment group received oral betamethasone (ATC H02AB01) at a dose of 3 mg once daily on days 1 and 2 followed by 2 mg once daily days 3, 4, and 5. Betamethasone intake was observed whilst admitted. After discharge, any remaining doses were given to the participant to take at home, but was not monitored. The dosing regimen is in line with recommendations from the Swedish Medical Products Agency for asthma exacerbations.^9^ Participants allocated to the control group received standard care excluding any adjuvant corticosteroid treatment.

Participants were discharged from the hospital at the discretion of the treating physician, with no pre-specified criteria. Follow-up assessments were conducted by phone on day 14, by in-person visit on day 28, and by phone on days 42, and 56.

Breathing rate, peripheral oxygen saturation (measured with pulse oximetry after 20 min of rest), oxygen use, pulse, blood pressure, body temperature, and mental state was monitored at least three times daily whilst admitted by non-study personnel on the ward and at the follow-up visit on day 28. Measurements were abstracted retrospectively from the charts. Medical records were searched to collect data on clinical variables including sex and medical imaging results prior to inclusion.

Participant-reported symptoms were followed using a structured questionnaire to identify the CAP score on days 1–7, 14, 28, 42, and 56 after inclusion. The CAP score was obtained per telephone or was self-reported after discharge. The CAP score is a validated disease-specific activity score for CAP ranging from 0 to 100, 0 marking the worst and 100 the best score.^10^ The CAP score is divided into a respiratory and a well-being domain.

Blood samples for analysis of C-reactive protein, white blood cell count, and haemoglobin were taken daily until normalised or discharge from hospital and on day 28. Fasting blood glucose were taken daily until discharge and on day 28.

Occurrence of harms or adverse events in the participants were assessed by study physician daily whilst admitted and per telephone or self-reported after discharge at days 1–7, 14, 28, 42, and 56, and elicited using the Common Terminology Criteria for Adverse Events version 4.0.

### Outcomes

The primary outcome was time from randomisation to resolution of hypoxaemia, defined as no longer requiring supplemental oxygen to maintain a peripheral oxygen saturation ≥93% measured by pulse oximetry after 20 min rest and a breathing rate of ≤20 breaths per minute, in an intention-to-treat analysis. The time of the first measurement where a participant had sustained resolution of hypoxaemia, defined as no subsequent oxygen requiring hypoxaemic episodes observed, was used as the end-point.

Secondary outcomes included time from randomisation to discharge from hospital, time from randomisation to resolution of fever, improvement in participant reported symptoms as determined by the CAP score questionnaire, and occurrence of adverse events.

### Statistical analysis

Power calculation was done using www.sealedenvelope.com. The significance level was set at 5% and the power at 90%. Based on a previous paediatric study, high-dose corticosteroids versus no corticosteroids resulted in defervescence by 48 h in 100% and 0%, respectively, and hypoxaemia lasted for a mean of <2 versus ∼3 days, respectively.^11^ The power calculation, therefore, assumed resolution of hypoxaemia by 48 h in 95% of participants with betamethasone and 65% of those without betamethasone. The sample size required in each group would then be 33. Assuming a 5% loss to follow up, 70 participants needed to be included. Based on this it would be possible to detect a hazard ratio of approximately 2.2.

Continuous variables were presented as medians (interquartile range [IQR]) and categorical variables as numbers (percentage). The values closest in time to randomisation were used for description of baseline characteristics. The level of hypoxaemia was described as the estimated partial pressure of oxygen in arterial blood (PaO_2_)/fraction of inspired oxygen (FiO_2_). PaO_2_ was estimated from SpO_2_ as described elsewhere.^12^

The primary outcome (time from randomisation to resolution of hypoxaemia) was analysed centrally using a Cox proportional hazards regression model in an intention-to-treat analysis and visualised with a Kaplan–Meier curve. A secondary per-protocol analysis was performed including participants who were compliant to the treatment protocol. Loss to follow up was censored at the time of loss to follow up. Results were presented as hazard ratios (HR) with 95% CI. The assumption of proportional hazards was assessed graphically by comparing curves on the log–log plot of survival. Any ties were handled by using the Breslow approximation. A post-hoc sensitivity analysis was performed with a model adjusted for estimated PaO_2_/FiO_2_ at baseline as a numerically higher count of participants in the control group received high flow oxygen treatment at randomisation. Moreover, post-hoc sensitivity analyses were performed with a models adjusted for symptom duration at inclusion and of treatment effects across pre- and post-pandemic periods by fitting a Cox regression model including an interaction term between treatment allocation and time period. Median time to resolution of hypoxaemia was estimated with the Laplace regression.^13^

The secondary outcomes, time to discharge from hospital, and time to resolution of fever were analysed in the same manner as the primary outcome. Improvement in participant-reported symptoms by the CAP score questionnaire was analysed using a linear mixed-effects model to address repeated measurement. Baseline CAP-score, treatment group, time, and treatment-by-time interaction were treated as fixed effects, with random intercepts for individual participants. A post-hoc analysis of decline of C-reactive protein day 1–7 was performed using a linear mixed-effects model in the same manner as for CAP-score.

The safety group was defined as any participants receiving at least one dose of betamethasone. Potential harms in the study were described by proportions of reported adverse events and any serious adverse event for participants in the safety group or not. Fasting blood glucose was monitored daily in participants during hospitalisation and at follow-up day 28. The proportion of participants experiencing blood glucose above the upper normal limit (>6.9 mmol/L) was registered. Differences between groups were analysed using Fisher's exact test.

A p-value <0.05 was considered significant for the primary outcome. Statistical analyses were performed using STATA/IC version 15.1 (StataCorp, TX). The trial did not include a data safety and monitoring board or interim analysis as the investigational medicinal product is well characterised at the doses used.

### Role of the funding source

The funder of the study had no role in study design, data collection, data analysis, data interpretation, or writing of the report.

## Results

**Fig. 1:**
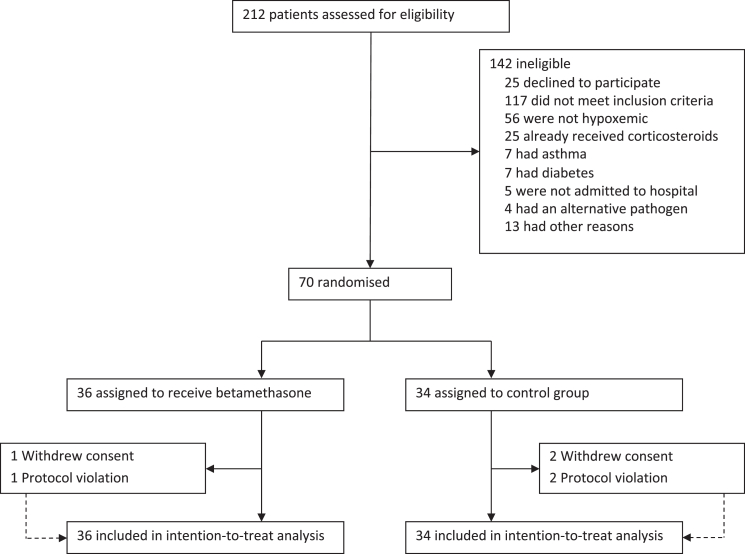
**Flowchart for study population**.

**Fig. 2:**
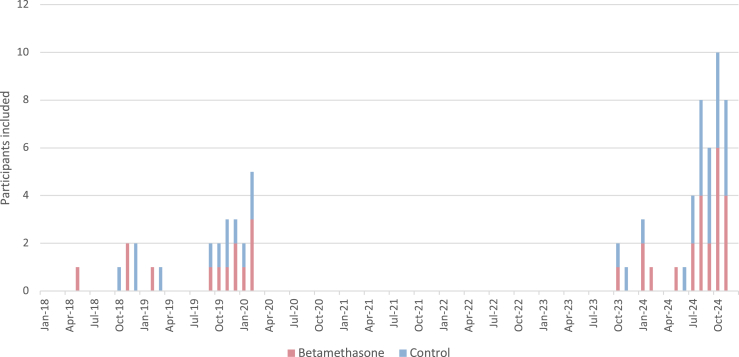
**Distribution of enrolled participants over time**.

From March 1, 2018, to November 14, 2024, a total of 212 patients were assessed for eligibility, and 70 participants (33%) were included. Of these, 36 were allocated to the betamethasone group and 34 to the control group. Reasons for exclusion are detailed in Fig. 1. No participants were included between March 2020 and September 2023 due to the effects of the SARS-CoV-2 pandemic, detailed in Fig. 2. The distribution of included participants for each site and PCR-assays used for detection of *M. pneumoniae* are detailed in Supplementary Table S1. Test results from microbiological sampling are presented in Supplementary Table S2. Of the 36 participants allocated to betamethasone treatment, one withdrew consent before any treatment was given, and one missed a single dose of the study drug. Of the 34 participants allocated to the control group, two withdrew consent during follow-up and another two received one or more doses of the study drug. Thus, 70 participants were included in the intention-to-treat analysis, 66 participants in the per-protocol analysis, and 37 participants, receiving at least one dose of betamethasone, in the safety group.

Baseline characteristics of the intention-to-treat population were similar between groups and are summarised in Table 1. The overall median age of the participants was 42 (IQR 29–49) years, and 57% (40/70) were males. A majority of participants (64% [45/70]) did not have any comorbidity. Twelve participants (three in the betamethasone group and nine in the control group) received high flow oxygen treatment on the day of inclusion. Another two participants in the control group were started on high flow oxygen treatment on day two from randomisation. No participant received invasive mechanical ventilation, and no participant died during the 56 days follow-up. Baseline data such as age, sex, respiratory rate, and estimated PaO_2_/FiO_2_ were well balanced between pre- and post-pandemic groups.

**Table 1:** Baseline characteristics at randomisation of the intention-to-treat population.

	Overall (n = 70)	Betamethasone group (n = 36)	Control group (n = 34)
Age, years	42 (28–49)	46 (32–51)	38 (28–46)
Male sex	40 (57)	19 (53)	21 (62)
Non-smoker	52 (74)	28 (78)	24 (71)
Current smoker	8 (11)	3 (8)	5 (15)
Previous smoker	10 (14)	5 (14)	5 (15)
No comorbidity	45 (64)	23 (64)	22 (65)
Hypertension	5 (7)	4 (11)	1 (3)
Ischaemic heart disease	0 (0)	0 (0)	0 (0)
Congestive Heart Failure	0 (0)	0 (0)	0 (0)
Chronic Kidney Disease	0 (0)	0 (0)	0 (0)
Pulmonary disease	0 (0)	0 (0)	0 (0)
Psychiatric disease	11 (16)	7 (19)	4 (12)
Body Mass Index (BMI), kg/m^2^	26 (23–31)	26 (23–30)	26 (22–32)
BMI <18.5	2 (3)	0 (0)	2 (7)
BMI 18.5–25	20 (34)	12 (41)	8 (28)
BMI 25–30	18 (31)	9 (31)	9 (31)
BMI >30	18 (31)	8 (28)	10 (34)
Symptom duration, days	11 (9–14)	11 (9–15)	11 (9–14)
Time from admission to randomisation, days	2 (2–3)	2 (2–3)	2 (2–3)
Antibiotic treatment prior to admission	46 (69)	22 (61)	24 (77)
Effective antibiotic treatment prior to admission	5 (7)	3 (8)	2 (6)
Macrolide treatment prior to inclusion	11 (16)	6 (17)	5 (15)
Respiratory Rate, breaths per minute	22 (19–26)	22 (20–26)	22 (18–26)
Estimated PaO_2_/FiO_2_	222 (158–270)	229 (182–272)	221 (142–266)
Body temperature, °C	37.3 (36.3–37.8)	37.3 (37.0–37.7)	37.3 (36.7–38.1)
Systolic blood pressure, mmHg	125 (115–134)	120 (114–130)	126 (119–135)
Diastolic blood pressure, mmHg	74 (69–83)	74 (70–84)	74 (65–80)
C-reactive protein, mg/L	137 (67–210)	139 (78–238)	128 (59–182)
Leucocyte count, × 10^9^/L	9.1 (7.1–11.7)	9.8 (6.6–12.0)	9.0 (7.9–11.1)
Lactate dehydrogenase, μkat/L	4.9 (4.2–5.7)	4.9 (3.9–5.5)	5.0 (4.6–6.0)
Fasting blood glucose, mmol/L	6.0 (5.5–6.8)	6.0 (5.6–7.0)	6.0 (5.4–6.7)
Bilateral pneumonia on medical imaging	37 (53)	21 (58)	16 (47)
Pleural effusion on medical imaging	8 (11)	2 (6)	6 (18)
Community-acquired pneumonia score	25 (13–36)	18 (12–36)	25 (17–36)
Respiratory score	19 (12–35)	17 (11–39)	21 (13–30)
Well-being score	21 (12–48)	21 (12–39)	37 (21–48)

Data are median (IQR) or number (%).

**Fig. 3:**
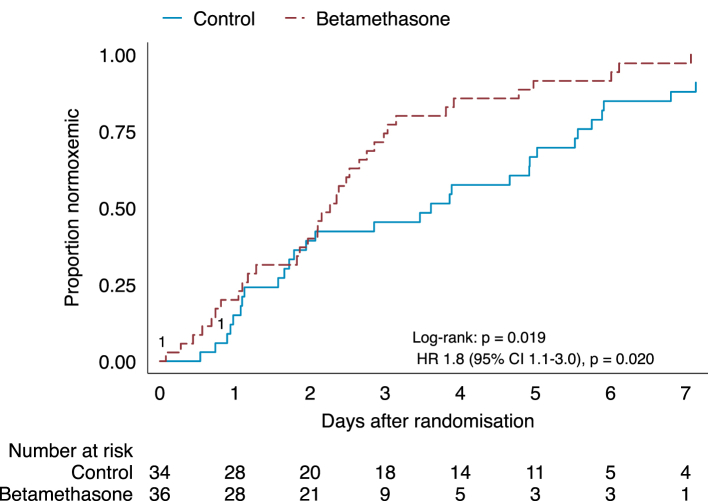
**Kaplan–Meier curve of time to resolution of hypoxaemia.** Resolution of hypoxaemia was defined as no longer requiring supplemental oxygen to maintain a peripheral oxygen saturation ≥93% measured by pulse oximetry after 20 min rest and a breathing rate of ≤20 breaths per minute.

Hypoxaemia resolved in all except two participants who withdrew consent before peripheral oxygen saturation normalised. Time to resolution of hypoxaemia was shorter in participants allocated to the betamethasone group (HR 1.82 [95% CI 1.10–3.02], p = 0.020) compared to the control group in the intention-to-treat analysis. The assumption of proportional hazards was graphically acceptable. The result was concordant in the per-protocol analysis (HR 1.75 [95% CI 1.05–2.92], p = 0.033) that included 34/36 and 31/34 participants from the betamethasone and control arms, respectively. A Kaplan–Meier curve of time to resolution of hypoxaemia is presented in Fig. 3. The estimated median duration of hypoxaemia was 2.3 (95% CI 1.8–2.7) days from randomisation in the betamethasone group and 3.6 (95% CI 1.9–5.3) days in the control group. *Post-hoc* analyses of time to resolution of hypoxaemia adjusted for estimated PaO_2_/FiO_2_ at inclusion and symptom duration were consistent with the primary analysis (Supplementary Tables S3 and S4). Moreover, the interaction term between treatment allocation and time period was not statistically significant (adjusted HR 0.70 [95% CI 0.25–1.90], p = 0.49) in the *post-hoc* analysis of treatment effects across pre- and post-pandemic periods, indicating that the treatment effect was similar between time periods (Supplementary Table S5).

The estimated median duration of hospitalisation was 2.9 (95% CI 2.3–3.4) days from randomisation for the betamethasone group and 3.9 (95% CI 2.2–5.6) days for controls (Supplementary Figure S1). The HR for time to discharge was 2.05 (95% CI 1.22–3.45, p = 0.007) for patients in the betamethasone group compared to controls in the intention-to-treat analysis. Duration of fever was not analysed as only 16 participants had fever at randomisation.

**Fig. 4:**
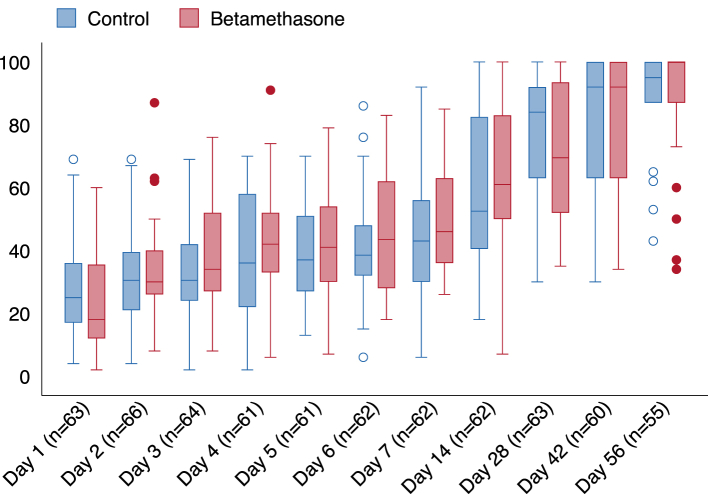
**Community-acquired Pneumonia (CAP) score over time.** Participant reported symptoms over time as measured by the CAP score were 0 marks the worst and 100 the best possible score for participants treated with betamethasone (red) and controls (blue). Outliers are visualised as circles.

Participant-reported symptoms as measured by the CAP score questionnaire were evaluated at a median of 11 (IQR 10–11) time-points for each participant. Improvement of CAP score did not differ (−0.09 units/day [95% CI −0.23 to 0.06], p = 0.24) between participants treated with betamethasone compared to controls (Fig. 4). Additionally, there were no group differences after dividing the CAP score in the respiratory (−0.07 [95% CI −0.24 to 0.11], p = 0.44) and well-being (−0.13 [95% CI −0.27 to 0.01], p = 0.06) domains (Supplementary Figures S2 and S3). C-reactive protein declined faster (−8.9 [95% CI −16.4 to −1.4] mg/L per day, p = 0.021) in the betamethasone group compared to controls over time the first seven days from inclusion.

Adverse events were evenly distributed between participants in the safety and control groups, as specified in Supplementary Table S6. A total of 33 adverse events were reported by 25 participants. The majority (29/33) were considered of mild or moderate severity, while four participants experienced a severe adverse event. None of the severe adverse events were considered to have a causal relationship with betamethasone treatment according to study physicians. A fasting blood glucose above the upper normal limit was reported in 16% (6/37) of the safety group and in 9% (3/33) of controls. Three participants in the safety group and one among the controls had a moderately elevated fasting blood glucose (>8.9–13.9 mmol/L), while the others were mildly elevated (>6.9–8.9 mmol/L).

## Discussion

To our knowledge, this is the first pathogen-specific, randomised controlled trial evaluating adjunctive corticosteroid treatment in hypoxaemic adults hospitalised with *M. pneumoniae* CAP. The trial demonstrated that adjunctive treatment with betamethasone shortened the time to resolution of hypoxaemia by approximately one day. The betamethasone regimen used was well tolerated and associated with clinically meaningful improvements for the participants and the healthcare system by shortening the need for oxygen treatment and hospital stay.

Supporting these results, a meta-analysis of 13 Chinese randomised trials including 1049 children with severe *M. pneumoniae* CAP reported that high-dose corticosteroids (10–30 mg/kg methylprednisolone) were associated with shorter length of stay and symptom duration compared to low-dose regimens (1–2 mg/kg methylprednisolone).^6^ In contrast, two large retrospective, propensity score matched cohorts of children (n = 51,633 and 885) reported an association between corticosteroid treatment and prolonged hospitalisation.^14^^,^^15^ Notably, corticosteroid doses used in paediatric studies were substantially higher than those used in the present trial, and data comparing corticosteroid treatment to no corticosteroids remain limited. Thus, extrapolation of paediatric findings to adult populations is problematic. Time to resolution of hypoxaemia was longer in the present study compared to the paediatric study using high-dose corticosteroids that the power calculation was based on. The moderate dose of corticosteroids given in the present study in addition to the differences of treating adults and children may account for the time difference.

Data on corticosteroid treatment of adults with *M. pneumoniae* infections are limited, with no prior randomised trials published. In contrast to the findings of the present study, two retrospective cohort studies (n = 2228 and n = 388) on adults hospitalised with *M. pneumoniae* CAP reported no benefit of corticosteroids on hospital length of stay.^7^^,^^8^ Neither was there an association between corticosteroid treatment and time to resolution of hypoxaemia.^8^ These studies employed measures to control for confounding such as propensity-score matching and regression models adjusted for potential confounders. However, residual confounding by indication, i.e. that patients treated with corticosteroids differed systematically from those not treated, remains a concern. This may explain why the reported results differ from the present randomised, controlled trial, that does not suffer from the same bias.

The pathophysiology of severe *M. pneumoniae* CAP is thought to involve an exaggerated host immune response.^16^ This may in part be mediated by the *M. pneumoniae* specific Community-acquired Respiratory Distress Syndrome (CARDS) toxin, which has been shown to induce extensive inflammation and immune-mediated lung injury.^17^^,^^18^ Moreover, a higher INF-γ response was reported in children with more severe *M. pneumoniae* CAP, indicating a T-cell mediated immunopathology.^19^ Corticosteroids have therefore been hypothesised to be particularly beneficial in *M. pneumoniae* CAP compared to other aetiologies of CAP. The shorter time to resolution of hypoxaemia in the treatment group in the present study supports this hypothesis. Importantly, no signs of rebound inflammation were observed following completion of the betamethasone course. Additionally, first-line antibiotics for *M. pneumoniae*, such as macrolides and tetracyclines have immunomodulatory properties that also ameliorate the immune response.^20^^,^^21^ In line with this, it was reported that the anti-inflammatory effects of tetracyclines were facilitated by inhibiting the inflammasome–caspase-1 pathway, leading improved survival in a murine model of acute lung injury.^22^ In the present study, antibiotic therapy was standardised to doxycycline, as this is the most commonly used antibiotic for *M. pneumoniae* infections in Swedish adults, and we wanted to evaluate the possible additional anti-inflammatory effects of corticosteroids.^1^ Given the lack of consensus on optimal antibiotic dosing and treatment duration and seeing that trial participants had severe pneumonia, we opted for a slightly higher dose and a longer treatment course than the Swedish standard.

Several randomised, controlled trials have evaluated the efficacy of adjunctive corticosteroids in patients with all-cause CAP. A meta-analysis of 15 trials including 3367 patients reported a reduction in 30-day all-cause mortality among patients with severe CAP treated with corticosteroids.^5^ In line with this, 30-day mortality was lower in a recent randomised, controlled trial including 2180 patients with CAP in a low-resource setting.^23^ However, these findings were not replicated in a randomised, controlled trial including patients with severe CAP (n = 658).^24^ Generalising results from studies on all-cause CAP to *M. pneumoniae* CAP is problematic as few patients (∼2%) with this specific pathogen were included. No fatalities occurred in the present study, consistent with the low in-hospital mortality (2.1% [28/1309]) reported in a recent French cohort.^25^ Moreover, no participants received invasive mechanical ventilation, compared to 4.8% (64/1309) in the French cohort. It is possible that patients on invasive mechanical ventilation at time of diagnosis were not included as they could not give informed consent and already might have received corticosteroid treatment in line with The Swedish Society of Infectious Diseases’ guidelines on CAP.^26^ Given the low mortality associated with *M. pneumoniae* CAP, mortality is not a feasible primary outcome in trials of this disease. Instead, as respiratory failure generally is the primary reason for hospitalisation, time to resolution of hypoxaemia was selected as the primary outcome in this study.

High-dose corticosteroids were associated with shorter length of stay and symptom duration compared to low-dose regimens in children with *M. pneumoniae* CAP.^6^ However, high-dose corticosteroids were associated with increased adverse events in adults with *M. pneumoniae* CAP.^7^ Given the typically non-fatal nature of *M. pneumoniae* infections, any adjunctive therapy should have a favourable safety profile. This study thus employed a moderately low dosing regimen of betamethasone in line with recommendations from the Swedish Medical Products Agency for asthma exacerbations.^9^ The long experience of comparable corticosteroid doses used for asthma and COPD exacerbations, and the lack of attributable adverse events, indicate that the betamethasone dose used is realistic for routine treatment of hypoxaemic *M. pneumoniae* CAP patients.^27^

Strengths of this trial include the randomised, controlled study design, as well as the pathogen-specific approach using a moderate betamethasone dose. However, the study has several limitations. The absence of a placebo and blinding may introduce bias, although the evaluation of the primary outcome by measuring peripheral oxygen saturation is relatively objective. It is conceivable that an unblinded nurse could have initiated earlier oxygen weaning and monitored oxygen saturation and respiratory rate more frequently in participants allocated to active therapy. However, we consider this unlikely, as both pulse oximetry measurements and oxygen titration were performed at the discretion of non-study clinical staff, and monitoring of oxygen saturation and respiratory rate generally is standardised. However, continuous peripheral oxygen saturation measurement on all participants could have provided more precise estimates. Diagnosis of *M. pneumoniae* infection was based on samples from either upper or lower respiratory tract reflecting standard clinical practice. This approach may, however, have introduced variability in how the inclusion criteria were applied. Moreover, even though patients were randomised, a numerically higher count of participants in the control group received high flow oxygen treatment, while bilateral infiltrates were more common in the treatment group. This may suggest that the proportion of Acute Respiratory Distress Syndrome differed between groups. A *post-hoc* regression analysis adjusted for PaO_2_/FiO_2_ at baseline was therefore performed and showed results consistent with the primary analysis. The relatively small sample size of this trial limits the precision of effect estimates and subgroup analyses, and a larger sample size may have facilitated a more balanced randomisation and more robust results. The study was interrupted by the SARS-CoV-2 pandemic. However, treatment effects were comparable between time-periods, and there were no significant group differences on important baseline characteristics between pre- and post-pandemically recruited participants. This is in line with large cohorts that reported no indication of increased proportions of post-pandemic severe disease in patients with *M. pneumoniae* infections.^4^^,^^28^ Finally, the study was a multi-centre trial conducted exclusively in Sweden, which may limit generalisability to other healthcare settings. Further studies are needed to investigate the potential benefit of corticosteroid treatment in patients with milder disease.

In conclusion, adjunctive treatment with a moderate and well-tolerated dose of betamethasone resulted in a clinically significant reduction of hypoxaemia duration in hypoxaemic adults with *M. pneumoniae* CAP in this open-label trial.

## Contributors

Conceptualization: KH, AN, JSJ, MH, JU. Methodology: KH, AN, MH, JU. Funding acquisition: KH, JU. Investigation: all. Project administration: KH. Supervision: JU. Data curation and verification: KH, JU. Formal analysis: KH. Writing—original draft: KH. Writing—review & editing: all. All authors had full access to all the data in the study and had final responsibility for the decision to submit for publication.

## Data sharing statement

Due to participant privacy reasons, participant level data cannot be shared.

## Declaration of interests

CJF report receiving payments for lectures for Tillots Pharma and Internetmedicin, outside of the submitted work. JSJ serves scientific advisory boards of Abbott, and Biomerieux and has received speakers’ honoraria from Hologic and Leo Pharma, outside of the submitted work. JT participates in one DSMB for an academic study of antibiotics in the intensive care unit. AN report receiving payments for lectures from Mediahuset, for expert testimony from IQVIA and Securis, for participating in Data Safety Monitoring Boards (DSMB) or Advisory Boards from Astra Zeneca, Pfizer, and Jansen Pharmaceuticals, and reimbursement for performing the E. mbrace study (Jansen Pharmaceuticals). All outside of the submitted work. JU report participating in DSMBs for two academic malaria studies, outside of the submitted work. No other authors report any conflicts of interest.

## Appendix A Supplementary data

Supplementary Appendix

Study protocol
