# Supplementary material for: Adjunctive betamethasone treatment of hypoxaemic adults hospitalised with Mycoplasma pneumoniae community-acquired pneumonia: an open-label, multicentre, randomised, controlled trial
Source: Lancet Reg Health Eur. 2026 Apr 19;64:101610. doi: 10.1016/j.lanepe.2026.101610 (PMC13147758; doi:10.1016/j.lanepe.2026.101610)
Supplement: Supplementary Appendix [file mmc1.pdf]

Supplementary Appendix

SUPPLEMENTARY APPENDIX..1

Supplementary Tables..2
Supplementary Table 1..2
Supplementary Table 2..3
Supplementary Table 3..4
Supplementary Table 4..4
Supplementary Table 5..4
Supplementary Table 6..5

Supplementary Figures..6
Supplementary Figure 1:..6
Supplementary Figure 2:..7
Supplementary Figure 3:..8

Supplementary Tables

Supplementary Table 1

**Supplementary Table 1. Number of Participants Enrolled and *M. pneumoniae* PCR-assays Used per Site**		
Study Hospital	***Participants included (n = 70)***	***M. pneumoniae PCR-assay***
Mälarhospital, Eskilstuna	2 (3)	P1 gene (LDT)[a]
Halmstad Hospital	1 (1)	P1 gene (LDT, BD Max)[b]
Skåne County	35 (50)	
Skåne University Hospital, Malmö	20 (29)	P1 gene (LDT)[a], Biofire Respiratory Panel RP2.1
Skåne University Hospital, Lund	8 (11)	P1 gene (LDT)[a], Biofire Respiratory Panel RP2.1
Helsingborg Hospital	7 (10)	P1 gene (LDT)[a], Biofire Respiratory Panel RP2.1
Stockholm County	29 (41)	
Danderyd University Hospital	27 (39)	Seegene Allplex™ Respiratory Panel 4, QIAstat-Dx Respiratory SARS-CoV-2 Panel
Saint Göran Hospital	2 (3)	P1 gene (LDT)[a]
Östersund Hospital	3 (4)	P1 gene (LDT)[a], VIASURE *C. pneumoniae*, *M. pneumoniae* & *L. pneumophila* Real Time PCR Detection Kit

Data are presented as number (%). Abbreviations: LDT, Laboratory Developed Test.
[a] Hardegger D et al. Rapid detection of Mycoplasma pneumoniae in clinical samples by real-time PCR. J Microbiol Methods 2000; 41:45-51.
[b] Unpublished

Supplementary Table 2

Supplementary Table 2. Microbiological Sampling of Participants

Treatment Group	Sample site Mp-PCR	Cq value Mp-PCR	Sputum culture, quantification (cfu/ml)	Nasopharyngeal culture	Respiratory panel (except *M. pneumoniae*)	Urinary antigen *Legionella*	Urinary antigen *Pneumococci*
**Control**	N	25.68	Neg.	Neg.	Neg.		
**Control**	N	29.57	Neg.	Neg.	Neg.	Neg.	Neg.
**Control**	N	19.23			Neg.	Neg.	Neg.
**Control**	N	23.77	Neg.		Neg.		
**Control**	N	37.0	Neg.	Neg.	Neg.	Neg.	Neg.
**Control**	N	33.93	Neg.		Neg.	Neg.	Neg.
**Control**	N	30.22		*M. catharralis*		Neg.	Neg.
**Control**	N	23.2	Neg.		Neg.		
**Control**	N	Pos.	Neg.	Neg.	Neg.		Neg.
**Control**	N	Pos.	Neg.	Neg.	Neg.		Neg.
**Control**	N	36.6		Neg.	Neg.		
**Control**	N	33.5		Neg.	Neg.	Neg.	Neg.
**Control**	N	27.9		Neg.	Neg.	Neg.	Neg.
**Control**	N	20.9	Neg.	Neg.	Neg.	Neg.	Neg.
**Control**	N	27.0		Neg.	Neg.		
**Control**	N/A	Pos.		Neg.	Neg.	Neg.	Neg.
**Control**	O	31.6		Neg.		Neg.	Neg.
**Control**	O	29.8		Neg.	Neg.		
**Control**	O	27.5		Neg.		Neg.	Neg.
**Control**	O	37.8		Neg.	Neg.	Neg.	Neg.
**Control**	O	27.2	Neg.	Neg.	Neg.	Neg.	Neg.
**Control**	O	25.1	Neg.		Neg.	Neg.	Neg.
**Control**	O	33.9		Neg.	Neg.	Neg.	Neg.
**Control**	O	25.8			Neg.		
**Control**	O	Pos.		Neg.	Neg.		
**Control**	S	26.89	Neg.		Neg.		
**Control**	S	16.36	Neg.		Rhinovirus		
**Control**	S	22.76	Neg.		Neg.		
**Control**	S	18.05	Neg.		Neg.	Neg.	Neg.
**Control**	S	19.7	Neg.				
**Control**	S	18.0					
**Control**	S	21.4		Neg.	Neg.	Neg.	Neg.
**Control**	S	20.1	Neg.		Neg.	Neg.	Neg.
**Control**	S	18.4		Neg.	Neg.		
**Betamethasone**	N	35.13			Neg.	Neg.	Neg.
**Betamethasone**	N	33.15	Neg.	Neg.	Neg.	Neg.	Neg.
**Betamethasone**	N	30.95	*β-haemolytic Streptococcus group C/G, (E5-E6)*	Neg.	Neg.		
**Betamethasone**	N	22.78	Neg.		Neg.		
**Betamethasone**	N	28.44			Neg.		
**Betamethasone**	N	28.91			Neg.	Neg.	Neg.
**Betamethasone**	N	38.11			Neg.		
**Betamethasone**	N	37.0			Neg.	Neg.	Neg.
**Betamethasone**	N	Pos.		*H. influenzae*	Neg.	Neg.	Neg.
**Betamethasone**	N	Pos.	*β-haemolytic Streptococcus group C/G, (E5-E6)*	Neg.	Neg.		
**Betamethasone**	O	Pos.	Neg.	Neg.	Neg.		
**Betamethasone**	O	Pos.	Neg.	Neg.		Neg.	Neg.
**Betamethasone**	O	26.7		Neg.	Neg.	Neg.	Neg.
**Betamethasone**	O	25.6		Neg.	Neg.		
**Betamethasone**	O	30.1		Neg.			
**Betamethasone**	O	27.6		Neg.	Neg.	Neg.	Neg.
**Betamethasone**	O	31.6		Neg.	Neg.	Neg.	Neg.
**Betamethasone**	O	23.5		Neg.	Neg.	Neg.	Neg.
**Betamethasone**	O	34.5		Neg.	Neg.	Neg.	Neg.
**Betamethasone**	O	29.2		Neg.	Neg.	Neg.	Neg.
**Betamethasone**	O	24.9		Neg.	Neg.	Neg.	Neg.
**Betamethasone**	O	Pos.	*H. influenzae, (E4)*		Neg.	Neg.	Neg.
**Betamethasone**	O/N (duo)	Pos.			Neg.	Neg.	Neg.
**Betamethasone**	O/N (duo)	Pos.			Neg.	Neg.	Neg.
**Betamethasone**	S	19.91	Neg.		Neg.	Neg.	Neg.
**Betamethasone**	S	16.7	Neg.		Neg.	Neg.	Neg.
**Betamethasone**	S	26.89			Neg.		
**Betamethasone**	S	15.84	Neg.	Neg.	Neg.		
**Betamethasone**	S	17.88	Neg.		Neg.	Neg.	Neg.
**Betamethasone**	S	15.76	Neg.		Neg.		
**Betamethasone**	S	14.15	Neg.	Neg.	Neg.	Neg.	Neg.
**Betamethasone**	S	15.15	Neg.		Neg.		
**Betamethasone**	S	17.3	Neg.		Neg.	Neg.	Neg.
**Betamethasone**	S	28.19	Neg.		Neg.	Neg.	Neg.
**Betamethasone**	S	20.8	Neg.			Neg.	Neg.
**Betamethasone**	S	19.2	Neg.		Neg.	Neg.	Neg.

Microbiological sampling of participants. Empty cells indicate that no sample was taken.
Abbreviations: Mp-PCR, Mycoplasma pneumoniae Polymerase Chain Reaction; Cq value, Cycle Quantification Value; Cfu, Colony-forming Unit; N/A, not available; N, nasopharynx; O, oropharynx; S, sputum; Neg., negative; Pos., positive.

Supplementary Table 3

Supplementary Table 3. Hazard Ratios for Regression of Hypoxemia, sensitivity analysis						
		Univariate			Multivariate	
**Variable**	**HR**	**95% CI**	***P* value**	**HR**	**95% CI**	***P* value**
Betamethasone	1.82	1.10-3.02	0.020	1.80	1.08-2.99	0.023
Estimated PaO_2/FiO_2	1.00	1.00-1.01	0.017	1.00	1.00-1.01	0.021

Hazard Ratios (HR) with 95 % Confidence intervals (95% CI) for regression of hypoxemia (defined as sustained $SpO_2 \geq$ 93% and respiratory rate ≤ 20 breaths/min without oxygen treatment) calculated by Cox regression. Sensitivity analysis adjusted for estimated PaO_2/FiO_2 at inclusion.

Supplementary Table 4

Supplementary Table 4. Hazard Ratios for Regression of Hypoxemia, sensitivity analysis						
		Univariate			Multivariate	
**Variable**	**HR**	**95% CI**	***P* value**	**HR**	**95% CI**	***P* value**
Betamethasone	1.82	1.10-3.02	0.020	1.81	1.09-3.00	0.022
Symptom duration (days)	0.98	0.93-1.04	0.59	1.00	0.93-1.05	0.68

Hazard Ratios (HR) with 95 % Confidence intervals (95% CI) for regression of hypoxemia (defined as sustained $SpO_2 \geq$ 93% and respiratory rate ≤ 20 breaths/min without oxygen treatment) calculated by Cox regression. Sensitivity analysis adjusted for symptom duration at inclusion.

Supplementary Table 5

Supplementary Table 5. Hazard Ratios for Regression of Hypoxemia, sensitivity analysis						
		Univariate			Multivariate	
**Variable**	**HR**	**95% CI**	***P* value**	**HR**	**95% CI**	***P* value**
Betamethasone	1.82	1.10-3.02	0.020	2.29	0.99-5.31	0.053
Post-pandemic inclusion	1.40	0.85-2.33	0.19	1.68	0.80-3.52	0.17
Betamethasone*Post-pandemic inclusion				0.70	0.25-1.92	0.49

Hazard Ratios (HR) with 95 % Confidence intervals (95% CI) for regression of hypoxemia (defined as sustained $SpO_2 \geq$ 93% and respiratory rate ≤ 20 breaths/min without oxygen treatment) calculated by Cox regression. Sensitivity analysis including pre- and post-pandemic time period and interaction-term between treatment and time period.

Supplementary Table 6

Supplementary Table 6. Detailed Reporting of Adverse Events			
Variable	***Betamethasone (n = 37)***	***Controls (n = 33)***	***P value***
Any adverse events	15 (41)	10 (30)	0.46
Severe adverse events†	2 (5)	2 (6)	1.0
Hyperglycaemia	6 (16)	3 (9)	0.49
Hyperglycaemia, >6.9-8.9 mmol/L	3 (8)	2 (6)	1.0
Hyperglycaemia, >8.9-13.9 mmol/L	3 (8)	1 (3)	0.62
Pulmonary embolism	1 (3)	0 (0)	1.0
Myopericarditis	0 (0)	1 (3)	0.47
Readmission for respiratory symptoms	1 (3)	0 (0)	1.0
Gastrointestinal disorder	3 (8)	4 (12)	0.70
Skin disorders	1 (3)	1 (3)	1.0
Fatigue	1 (3)	1 (3)	1.0
Dizziness	0 (0)	1 (3)	0.47
Swollen tonsils	1 (3)	0 (0)	1.0
Headache	1 (3)	1 (3)	1.0
Agitation	1 (3)	0 (0)	1.0
Urinary retention	0 (0)	1 (3)	0.47
Tachycardia	0 (0)	1 (3)	0.47
Depression	1 (3)	0 (0)	1.0
Thrombophlebitis	0 (0)	1 (3)	0.47
Rib fracture	1 (3)	0 (0)	1.0

Data are presented as number (%). P values calculated with Fisher's exact test.

† Including one case each of pulmonary embolism, myopericarditis, readmission for respiratory symptoms, and fatigue.

Supplementary Figures

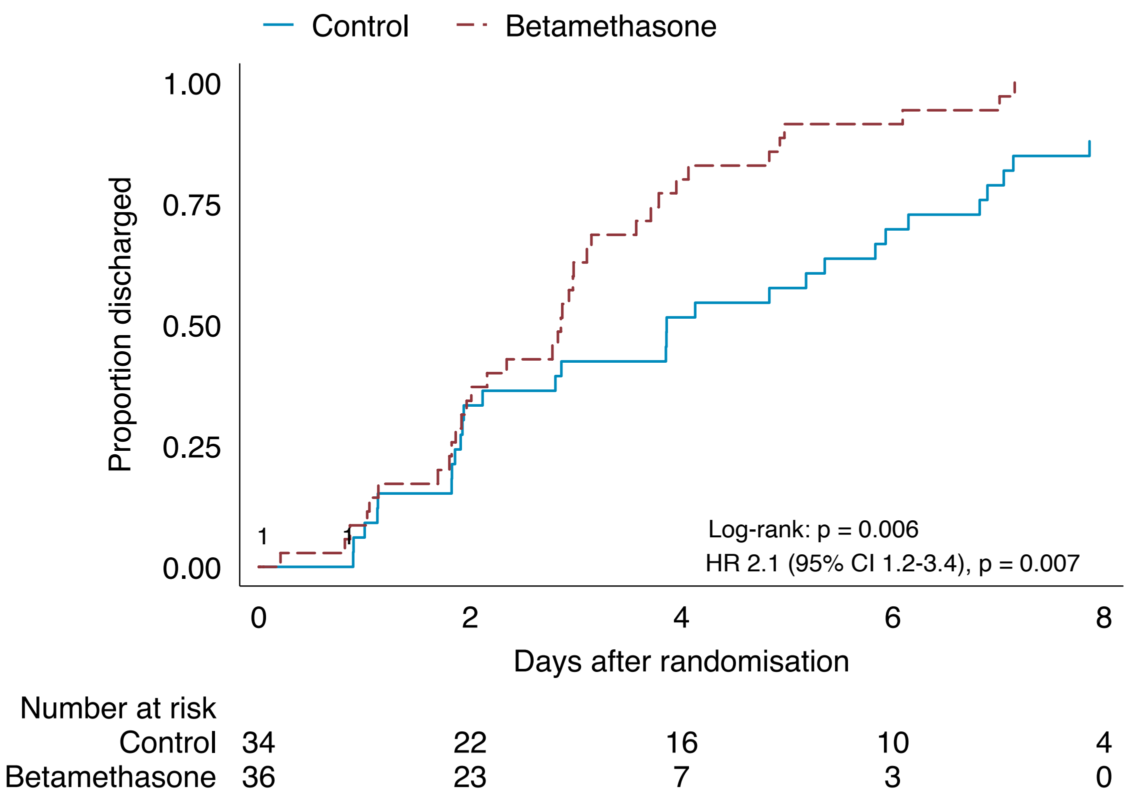

Supplementary Figure 1: **Kaplan-Meier Curve of Length of Stay.**

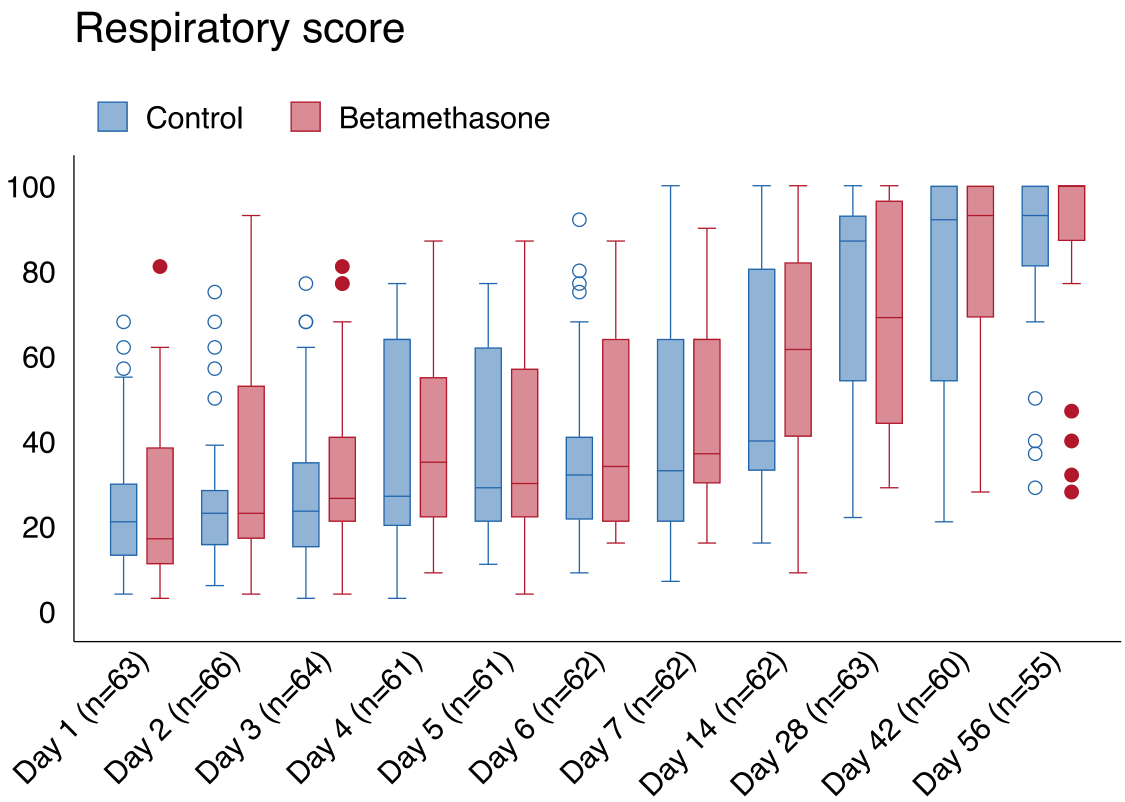

Supplementary Figure 2: **Community Acquired Pneumonia (CAP) Respiratory Score Over Time.** Participant reported symptoms over time as measured by the CAP score's respiratory domain were 0 marks the worst and 100 the best possible score for participants treated with betamethasone (red) and controls (blue). Outliers are visualised as circles.

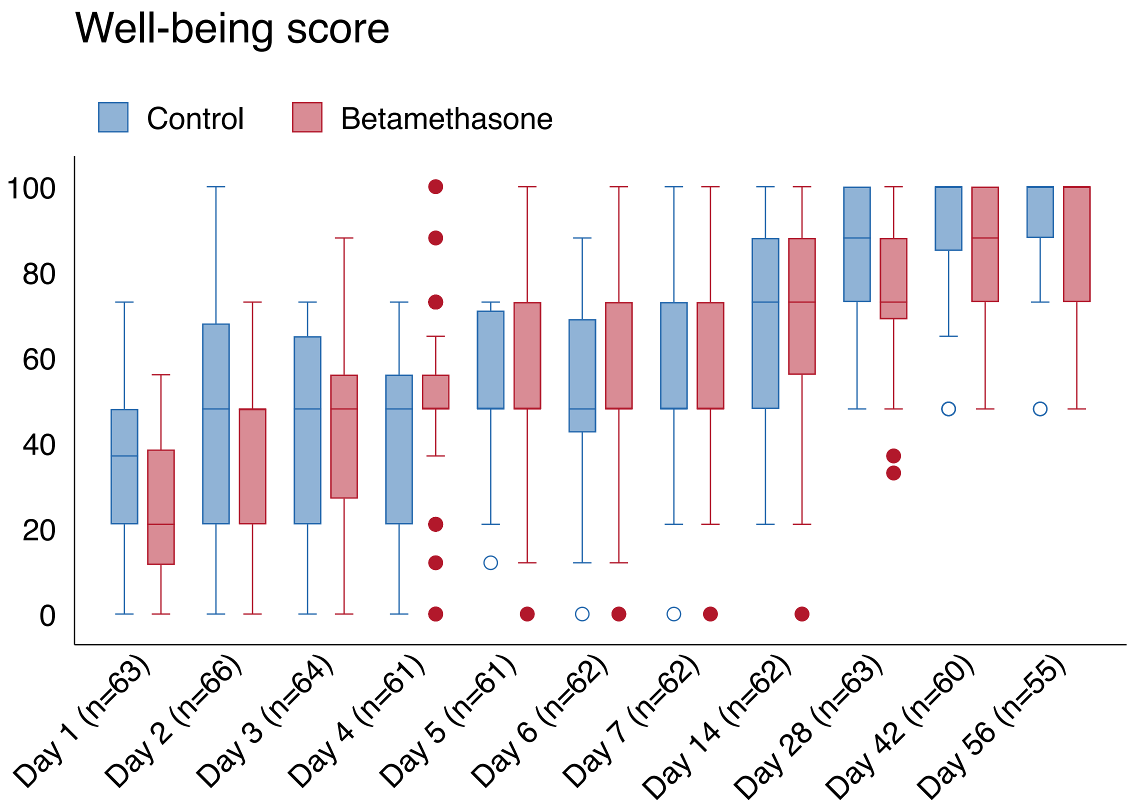

Supplementary Figure 3: **Community Acquired Pneumonia (CAP) Well-being Score Over Time.** Participant reported symptoms over time as measured by the CAP score's well-being domain, were 0 marks the worst and 100 the best possible score for participants treated with betamethasone (red) and controls (blue). Outliers are visualised as circles.
