# Supplementary material for: Adjunctive betamethasone treatment of hypoxaemic adults hospitalised with Mycoplasma pneumoniae community-acquired pneumonia: an open-label, multicentre, randomised, controlled trial
Source: Lancet Reg Health Eur. 2026 Apr 19;64:101610. doi: 10.1016/j.lanepe.2026.101610 (PMC13147758; doi:10.1016/j.lanepe.2026.101610)
Supplement: Study protocol [file mmc2.pdf]

A randomised, multicentre, controlled trial to compare the duration of supplemental oxygen treatment in adults with *Mycoplasma pneumoniae* pneumonia treated with betamethasone or not, in addition to antibiotics

EudraCT number 2016-002585-32
Sponsor protocol number mpp01
Version number 3.3
2018-10-16

Signatures

Sponsor and national coordinating investigator

Johan Ursing

2018 1019
Date

Table of contents

List of used abbreviations 4
Contact information 4
Synopsis 6
Background 7
Aim 7
Rationale 8
Benefit-risk assessment 8
Outcomes 8
Primary endpoint: 8
Secondary endpoints: 8
Methods 9
Study Design 9
Study population 9
Inclusion criteria: 9
Exclusion criteria: 9
Patient screening and patient identification logs: 10
Study Procedures 10
Randomisation: 10
Treatment: 10
Monitoring and follow up: 11
Comorbidities: 12
Laboratory Procedures 12
Biobank 13
Safety and adverse events 13
Specification of safety parameters 13
Adverse event 14
Definition: 14
Clarifications: 14
Serious adverse event 15
Definition: 15

Rating scales ... 15
Intensity: ... 15
Causality: ... 15
Reporting procedures for serious adverse events ... 16
Reporting period for adverse events ... 16
Sponsor's reporting of serious adverse drug reactions and suspected unexpected serious adverse reaction ... 16
Stopping rules ... 17
Study management ... 17
Independent Data Safety and monitoring board (DSMB) ... 17
Clinical monitoring ... 17
Audits and inspections ... 18
Training of study personnel ... 18
Changes to the study protocol ... 18
Protocol deviations ... 19
Study reporting ... 19
Archiving ... 19
Patient confidentiality ... 19
Insurance ... 19
Publication ... 19
Quality control (QC) and quality assurance (QA) ... 20
Ethical considerations ... 20
Statistics ... 20
Power calculation: ... 20
Duration: ... 21
Data storage: ... 21
Data analysis: ... 21
References ... 22

List of used abbreviations

MPA Medical Products Agency
SmPC Summary of Product Characteristics
CRF Case Record forms
SAR Serious adverse event
SUSAR Suspected unexpected adverse event
CSP Clinical Study Protocol

Contact information

Sponsor: Johan Ursing, M.D., Associate professor, Department of Infectious Diseases, Danderyd Hospital, Danderyd, Sweden

Investigators:	E-mail:	Telephone:
Akademiska sjukhuset, Uppsala		
Karolina Nissen M.D.	Karolina.nissen@akademiska.se	018-6110000
Capio S:t Görans sjukhus		
Milena de Oliveira e Costa, M.D.	Milena.Costa@capiostgoran.se	08-58701000
Danderyds Sjukhus, Danderyd		
Johan Ursing M.D., PhD.	Johan.Ursing@gmail.com	0704751530
Karl Hagman M.D.	Karl.Hagman@sll.se	0739592027
Hallands sjukhus Halmstad		
Johan Ljungberg M.D.	Johan.ljungberg@regionhalland.se	035-131000
Helsingborgs lasarett		
Cecilia Rydén, M.D., PhD.	Cecilia.ryden@med.lu.se	042-4061000
Mälarsjukhuset, Eskilstuna		
Göran Stenlund M.D.	Göran.stenlund@dll.se	016-103000
Norrlands Universitetssjukhus, Umeå		
Maria Furberg, M.D, PhD.	maria.furberg@umu.se	090-7850000
Sjukhuset i Gävle		
Lena-Maria Söder M.D.	lena-maria.johansson.soder@regiongavleborg.se	026-154000
Skaraborgs Sjukhus, Skövde		
Marianne Schlaug M.D.	Marianne.Schlaug@vgregion.se	0500-431000
Skånes Universitetssjukhus, Lund		
Carl-Johan Fraenkel M.D.	Carl-Johan.fraenkel@skane.se	046-171000

Skånes universitetssjukhus, Malmö
Anna Nilsson M.D., PhD. Anna.Nilsson@med.lu.se 040-337760

Södersjukhuset, Stockholm
Richard Dwyer M.D., PhD. Richard.Dwyer@sll.se 08-6161000

Universitetssjukhuset i Linköping
Jakob Paues, M.D., PhD. jakob.paues@regionostergotland.se 010-1030000

Östersunds sjukhus
Elin Hedman M.D, elin.hedman@regionjh.se 063-153000
Karin Biasoletto M.D. karin.biasoletto@regionjh.se 063-153000

Karolinska Institutet
Piotr Nowak M.D., PhD piotr.nowak@ki.se 08-58580000

Monitor:

Infektionskliniken, Danderyds Sjukhus
Magnus Hedenstierna M.D., PhD. Magnus.Hedenstierna@sll.se 08-12355000

Synopsis

Mycoplasma pneumoniae is one of the leading causes of community acquired pneumonia. Although normally self-limiting, a proportion of patients develop more severe infection and have to be admitted to hospital. Available data suggests that corticosteroids are beneficial for severe *M. pneumoniae* pneumonia in children and for all-cause community acquired pneumonia. However, the corticosteroid doses tested are high and data on adults admitted to hospital with *M. pneumoniae* pneumonia is lacking. The aim of this randomised, controlled, multicentre trial is to determine if oral intake of 12 mg (total dose) of betamethasone over 5 days, as an adjunct to antibiotics, is beneficial for the treatment of adults hospitalised with *M. pneumoniae* pneumonia. The primary endpoint of this study is time to resolution of hypoxemia. Secondary endpoints include time to discharge from hospital, improvement in subjective well-being, shortness of breath and cough as determined by a standardised questionnaire, time to resolution of fever, impact on eradication of *M. pneumoniae* and identifying inflammatory fingerprints for prediction of severe *M. pneumoniae* infection. We assume resolution of hypoxemia by 48 hours in 95% of participants with betamethasone and 65% of those without betamethasone. In order to detect this with 90% power, 70 adult patients hospitalised with *M. pneumoniae* pneumonia at any of our study hospitals during the course of two years will be included. EudraCT number 2016-002585-32.

Background

Mycoplasma pneumoniae is one of the leading causes of community acquired pneumonia. Respiratory symptoms are typically dominated by cough. *M. pneumoniae* pneumonia is normally self-limiting, however cough and fatigue may last for many months and be quite debilitating (1). A proportion of patients develop more severe infection and have to be admitted to hospital. Characteristics of these patients are younger age, fewer comorbidities and lower mortality compared to other causes of community acquired pneumonia (2). Despite being young and with no comorbidities these patients tend to have high breathing rates and considerable oxygen demand resulting in hospitalisation.

The severity of *M. pneumoniae* pneumonia appears to be a dependent upon the immune response through various mechanisms including an allergic reaction as wells as the bacterial load (2). In line with this, corticosteroids have been reported to be beneficial in children with refractory *M. pneumoniae* pneumonia. Three case series using high doses of corticosteroids reported rapid improvement (3-5). A randomised trial (n=58) in which the treatment group received 1 mg/kg oral prednisolone twice daily for 5 days in addition to azithromycin found that the duration of fever, hypoxemia and dyspnoea were shorter in the treatment group. Resorption of pulmonary infiltrates and atelectasis were also faster (6). These patients had refractory *M. pneumoniae* pneumonia defined as prolonged fever and deterioration of clinical and radiological findings after 7 days of azithromycin treatment. Another study randomised children admitted for *M. pneumoniae* pneumonia to prednisolone treatment within 24 or 72 hours and found that fever duration, hospital stay and radiographic resolution were faster with earlier treatment (7).

A recent study found 50 mg of prednisolone daily for 7 days to be beneficial for treatment of adults with all-cause community acquired pneumonia (8). Asthma is typically treated with 30-60 mg prednisolone or 4-8 mg betamethasone daily until improvement or for 10 days (9). However, studies on corticosteroid use in adults hospitalised with *M. pneumoniae* pneumonia are lacking.

Aim

The aim is to determine if corticosteroid treatment, as an adjunct to antibiotics, is beneficial for the treatment of adults hospitalised with *M. pneumoniae* pneumonia.

Rationale

Data and clinical impression suggests that much of the illness associated with *M. pneumoniae* pneumonia is driven by inflammation and consequently corticosteroid treatment should be beneficial. Available data suggests that corticosteroids are beneficial for severe *M. pneumoniae* pneumonia in children and for all cause community acquired pneumonia in adults. However, the corticosteroid doses used are high and data on adults admitted to hospital with *M. pneumoniae* pneumonia is lacking. Based on studies in children the potential benefits are faster symptom resolution and shorter hospital admittance.

Benefit-risk assessment

In this study we will use betamethasone (ATC H02AB01) given orally according to the Summary of Product Characteristics (SmPC) in a dose that is expected to be well tolerated. Possible adverse events of this treatment include hyperglycemia, hypertension, gastric ulcers and mood changes amongst others. Blood samples taken in the study may be painful, but are not harmful. Possible benefits include shorter time to recovery and the extended follow-up will enable a more rapid detection of possible treatment failures. Additionally, the risk of developing severe mycoplasma pneumonia requiring intensive care treatment is likely to be reduced in future patients should betamethasone treatment prove to be effective. We therefore deem the potential benefits of this study superior to the potential risks.

Outcomes

Primary endpoint: Time to resolution of hypoxemia defined as no longer requiring supplemental oxygen to maintain a peripheral oxygen saturation above 92% measured with pulse oximetry after 20 minutes rest and a breathing rate of ≤20 breaths per minute.

Secondary endpoints: Secondary endpoints include time to discharge from hospital, improvement in subjective well-being, shortness of breath, and cough as determined by CAP score questionnaire, time to resolution of fever, impact on eradication of *M. pneumoniae* and identifying inflammatory fingerprints for prediction of severe *M. pneumoniae* infection.

Methods

Study Design

An open, randomised, controlled, multicentre, clinical trial conducted at the following hospitals in Sweden: Akademiska sjukhuset (Uppsala), Capio S:t Görans sjukhus (Stockholm), Danderyds sjukhus (Stockholm), Hallands sjukhus Halmstad, Helsingborgs lasarett, Mälarsjukhuset (Eskilstuna), Norrlands Universitetssjukhus (Umeå), Sjukhuset i Gävle, Skaraborgs sjukhus Skövde, Skånes Universitetssjukhus Lund, Skånes Universitetssjukhus Malmö, Södersjukhuset (Stockholm), Universitetssjukhuset i Linköping and Östersunds sjukhus. The study will be conducted in accordance with the Helsinki declaration on ethical principles for medical research involving human subjects.

Study population

Patients with *M. pneumoniae* pneumonia admitted to a study hospital will be considered for study entry. Patients will be identified by notification from the respective hospitals Microbiological Laboratory and by referral from colleagues. Patients will be informed of the study and offered to participate if they match inclusion and exclusion criteria. Upon inclusion case record form A will be filled in (appendix 1) and the informed consent form will be signed.

Inclusion criteria: Age ≥18 years, active *M. pneumoniae* pneumonia defined as chest x-ray or CT-scan showing an infiltrate and positive *M. pneumoniae* PCR on sample taken from upper or lower airways, admitted to a study hospital, hypoxemia defined as having a peripheral oxygen saturation below 93% (measured by pulse oximetry) and a breathing rate of >20 breaths per minute without supplemental oxygen treatment, a negative pregnancy test taken before inclusion and usage of an acceptable effective method of contraception until treatment discontinuation if the participant is a woman of childbearing potential, written informed consent after meeting with a study physician and ability and willingness to complete follow up.

Exclusion criteria: Significant growth of alternative lower airway pathogen such as *Streptococcus pneumoniae* or *Haemophilus influenzae* in sputum, known current gastric ulcer, pregnancy, breast feeding, diabetes mellitus, chronic obstructive airway disease, asthma, hypersensitivity to any ingredient in the bethamethasone, inability to give informed consent or significantly compromised immunity. Compromised immunity includes but is not limited to

treatment with major immunosuppressive agents including high dose corticosteroids, anti-TNF agents, calcineurin inhibitors, mTOR inhibitors, lymphocyte depleting biological agents, chemotherapeutic anti neoplastic agents. Also patients with advanced HIV/AIDS, severe immunodeficiency such as hypoglobulinemia, decompensated liver cirrhosis and bone marrow transplant the last year will be excluded.

Patient screening and patient identification logs: Investigators will keep a record of all patients that are considered for enrolment even if they are not subsequently enrolled in order to verify that the patient population was selected without bias (ICH-GCP 8.3.20). The reasons for non-eligibility are to be defined in terms of one or more of the eligibility criteria.

Study Procedures

Randomisation: All participants will be randomised to either a treatment group or a control group (A or B) using www.sealedenvelope.com. Randomisation is blocked (using random permuted blocks) with 1:1 allocation ratio to ensure that the groups are balanced periodically.

Treatment: All participants will take doxycycline (ATC J01AA02) 200 mg once daily until 10 days total effective treatment has been taken. If participants have been treated with active antibiotics before inclusion, treatment will be switched to doxycycline upon inclusion. Total effective antibiotic treatment duration will be 10 days. The doxycycline dose chosen is slightly higher than normal as the doxycycline minimal inhibitory concentration is very similar to that obtained when 100 mg is given daily. The treatment duration is ten days as this group of patients have complicated mycoplasma pneumonia.

Participants randomised to treatment with betamethasone will take 3 mg once daily days 1 and 2 and 2 mg once daily days 3, 4 and 5, given orally according to the SmPC. Betamethasone intake will be observed whilst admitted and confirmed by questioning thereafter. After discharge, any remaining doses will be labelled with dose information as done in standard clinical practice and given to the patient to take at home.

The betamethasone dose has been selected based on clinical experience, similarity to that used for treatment of exacerbations of chronic obstructive pulmonary diseases and asthma, tolerability and likely acceptance by clinical practitioners.

Betamethasone (ATC H02AB01) is a corticosteroid with a Marketing Authorization in Sweden (MA number 6940). It will be provided free of charge to participants and taken from

the pharmaceutical store room of the ward. As the drug only is administered directly from the ward, no study specific marking - except dose to be taken - is needed.

Patients randomised to the control group will only receive standard care with doxycycline as described above.

Monitoring and follow up: Whilst admitted breathing rate, peripheral oxygen saturation (measured with pulse oximetry after 20 minutes of rest), oxygen use, pulse, blood pressure, body temperature, peak expiratory flow and mental state will be monitored three times daily and entered into computerised medical records.

Medical records will be searched to determine values prior to inclusion in the study and data will be entered on to clinical records form B (appendix 2). Chest x-rays or CT scan will have been performed routinely prior to inclusion.

Following inclusion, participants will also be followed up with structured questionnaires (appendix 3, 4 and 5) to identify the community acquired pneumonia (CAP) score on days 1, 2, 3, 4, 5, 6, 7, 14, 28, 42 and at 2 months after inclusion. If the patient has been discharged, physiological parameters will not be recorded but the CAP score will be obtained per telephone. The CAP score is a disease-specific activity score for community acquired pneumonia; it ranges from 0 to 100, 0 marking the worst and 100 the best score (10).

Blood samples for analysis of C-reactive protein (CRP), white blood cell count (WBC) and haemoglobin (Hb) will be taken daily until normalised or discharge from hospital and on day 28. Fasting blood glucose and blood samples for immunological analyses will be taken daily until discharge and on day 28. Blood samples for *M. pneumoniae* serology will be taken on the day of inclusion and at day 28. A sample for diagnostic PCR and for subsequent phenotypic and genotypic characterisation of *M. pneumonia* bacteria will be taken from upper airways prior to inclusion, upon inclusion and on day 28. Samples will be frozen at -80°C until phenotypic and genotypic characterisation. Patients will be discharged from the hospital at the discretion of treating physician. Follow up visits will be done on day 28 and by phone on days 42 and 56.

Table 1. Planned questionnaire and sampling follow up

Time point (days)	Prior to or upon inclusion	Daily whilst admitted	7	14	28	42	56
**Clinical records form A**	X						
**Clinical records form B/C**		X[1]	X	X	X	X	X
**Sampling**							
**CRP, WBC, Hb**	X	X[2]			X		
**Fasting blood-glucose**		X			X		
**Immunological analyses**		X			X		
***M. pneumoniae* serology**	X[3]				X		
**Nasopharyngeal sample for multivirus PCR**	X[3]						
**Upper airway sample[4]**	X				X		
**Chest X-ray**	X						

[1] CAP score will be obtained via telephone day 1-6 if the patient has been discharged

[2] Laboratory parameters will be taken daily until normalised

[3] *Mycoplasma pneumoniae* serology and upper airway sampling will be taken on day of inclusion

[4] Upper airway sample for diagnostic *M. pneumoniae* PCR and for subsequent phenotypic and genetic *M. pneumoniae* characterisation

Comorbidities: Will be obtained from the patient and medical records and recorded in clinical records form A. On the day of inclusion a nasopharyngeal aspirate will be taken for analyses of potential co-infection with Influenza A+ B, Parainfluenza 1, 2, 3, Human Bocavirus, Human Metapneumovirus, Coronavirus HKUL, OC43, 229E, NL63, Adenovirus, Enterovirus, Rhinovirus and Respiratory Syncytial virus.

Laboratory Procedures

Analyses of CRP, WBC, Hb, PCRs and serologies will be done at respective hospitals affiliated Laboratory.

Upper airway samples for phenotypic and genotypic characterisation of *M. pneuminae* will be stored in Universal Transport Medium and frozen at -80C at respective study site. Prior to analysis samples will be collected at irregular intervals and stored centrally at Karolinska Institutet biobank.

Fasting blood glucose values will be analysed on the wards whilst admitted.

Daily whilst admitted and at the day 28 follow up, 15 ml blood samples (10 ml EDTA tube and 5 ml tube without anticoagulants) will be obtained. The EDTA blood will be processed by ficoll centrifugation; obtained PBMC and plasma will be stored at liquid nitrogen and -70C respectively. The serum obtained from 5 ml blood (sampled without anticoagulant) will be stored at -70C.

Soluble factors such as cytokines and chemokines will be evaluated using immunoassays (LUMINEX, ELISA). Flow cytometry will be utilized to evaluate the cellular immune activation (T cells and monocytes). The cellular and soluble immune activation markers will be compared with clinical metadata to reveal the association with disease severity and clinical parameters.

Biobank

Microbiological samples and blood samples will be stored as part of the Karolinska Institutet biobank.

Safety and adverse events

Upon inclusion an entry will be made in the patient's medical record specifying that the subject is participating in this study, if betamethasone treatment is given, and that written informed consent is obtained.

Specification of safety parameters

Safety parameters; Adverse drug reactions and serious adverse drug reactions and changes in vital signs.

Any apparent side effects experienced by the subject will be assessed from the time the subject signs the informed consent and as long as the subject is part of the study, and will be reported by study site personnel either as a Baseline Event or an AE. The study personnel will document any Baseline Events or AEs in the CRF, whether observed by the investigator or reported by the subject. The safety of the different treatment arms will be assessed with regard to AEs, baseline medical conditions, and findings from the physical examination and laboratory tests.

The Betamethasone dose used is expected to be well tolerated. Possible side effects include hyperglycemia, hypertension, gastric ulcers and mood changes such as confusion and agitation amongst others. For a full list of possible side effects from betamethasone treatment see the attached SmPC.

Possible adverse events will be elicited using a modification and Swedish translation (appendix 6) of Common Terminology Criteria for Adverse Events v4.0 (11) and they will be continuously reported to the sponsor. Adverse events related to betamethasone treatment shall be followed to assess reversibility.

An annual Development Safety Update Report (DSUR) will be sent to the Swedish Medical Products Agency and the Stockholm Regional Ethical Review Board.

Adverse event

Definition: An AE in this study is defined as any untoward medical occurrence in a study subject. The occurrence does not necessarily need to have a causal relationship with the Investigational Medicinal Product (IMP). An AE can therefore be any unfavourable and unintended sign (including an abnormal laboratory finding), symptom, or disease temporally associated with the study, whether or not causally related to administration of the IMP.

The occurrence of an AE may come to the attention of study personnel during study visits and interviews of a study recipient presenting for medical care, or upon review by a study monitor who is scrutinising relevant source data.

Clarifications: Diagnostic and therapeutic non-invasive and invasive procedures, such as surgery, should not be reported as AEs. However, the medical condition for which the procedure was performed should be reported if it meets the definition of an AE. For example, an acute appendicitis that begins during the AE reporting period should be reported as "acute appendicitis" and the resulting appendectomy noticed under Comments. Pre-study conditions, which led to elective surgery during the time of the study, are not to be reported as AEs.

If an abnormal laboratory value or vital sign is associated with corresponding clinical signs and symptoms, the sign/symptom should be reported as the AE and the associated laboratory

result or vital sign should be considered additional information that is to be collected in the CRF.

Serious adverse event

Definition: An SAE in this study is defined as any untoward medical occurrence that meets one of the following criteria:

- Results in death
- Is life threatening (the term "life threatening in the definition of "serious" refers to an event in which the subject was at risk of death and the time of the event; it does not refer to an event which hypothetically might have caused death if it was more severe)
- Requires inpatient hospitalization or prolongation of existing hospitalization
- Results in persistent or significant disability/incapacity
- Is an important medical event

Rating scales

The investigator is to record all directly observed AEs and all AEs spontaneously reported by the subject in the subject's medical records (source data) and in the CRFs using concise medical diagnostic terminology. All AEs must be graded for:

- seriousness
- intensity
- causality (possible relationship) to the IMP

The question asked will be "Have you had any health problems since your last evaluation?" If no AE has occurred during the period concerned, this should actively be noted in the CRF.

Intensity: The expression intensity of adverse events means the intensity of the event in the opinion of the subject. The intensity of each AE is to be graded by the Investigator.

Causality: The relationship between the IMP and each AE has to be classified by the Investigator using one of the following terms:

Unlikely: The onset of the AE and administration of the IMP are such that the medication is not likely to have any reasonable association with the AE.

Possible: It might be possible that the AE could have been caused by the IMP.

Probable: It is probable that the AE is caused by the IMP.

Reporting procedures for serious adverse events

Events deemed as serious must be reported to the sponsor within 24 hours after the Investigator's awareness of the SAE. This short time frame is in compliance with international regulations. All SAEs should be reported by the site staff in the CRF.

If the initial report is not complete, it should be followed by submission of a more detailed report within five calendar days.

All SAEs must be followed until resolution or until the Investigator assesses them as being under full control.

The reporting period for SAEs starts at betamethasone administration and ends at the final follow-up visit 2 months after end of IMP administration.

Reporting period for adverse events

All AEs, irrespective of nature, will be followed until resolution or until end of the follow-up period.

The reporting period for AEs starts at inclusion and ends at the final follow-up visit 2 months after inclusion.

Sponsor's reporting of serious adverse drug reactions and suspected unexpected serious adverse reaction

The investigator or designee have to make a causality (relationship) assessment. The term SADR (Serious Adverse Drug Reaction) is to be used when the Investigator deems the SAE as possibly or probably related to the IMP. Serious Adverse Drug Reactions are to be reported annually to the MPA and the ethical committee.

If the event is not described before, e.g. in the SmPC, the event is a SUSAR (Suspected Unexpected Serious Adverse Reaction). The sponsor has the obligation to submit a SUSAR report to the Swedish Medical Products Agency (via a CIOMS-form) and to the Stockholm Regional Ethical Review Board within:

- 7 days if fatal or life-threatening (follow-up information within an additional 8 days)
- 15 days if non-fatal and non-life-threatening (follow-up information as soon as possible)

The sponsor has the obligation to, once a year throughout the clinical study (or on request); submit a safety report to the MPA and the IEC taking into account all new available safety information received during the reporting period.

Stopping rules

If an attending physician assesses that a patient is suffering from an adverse event induced by the IMP and requiring treatment, IMP treatment will be stopped for that patient. The outcome will be classified as an adverse event necessitating treatment stop.

A study subject can terminate his/her participation in the study at any time without giving a reason why. Should a subject wish to do so he/she will be treated in line with standard recommendations.

Study organisers can end a patient's participation for safety reasons.

If a patient is excluded, already collected data will be used in subsequent analyses unless he/she objects.

Study management

Independent Data Safety and monitoring board (DSMB)

No DSMB is required as the IMP is safe and well characterised at the dose used and as the study is of short duration, not blinded, the subjects are adults and are not suffering from a life threatening illness.

Clinical monitoring

Before the initiation of the study, the monitor will:

- Determine the adequacy of the facilities.
- Discuss with the investigator and study personnel their responsibilities with regard to CSP adherence, local regulations, and the duties of monitor.
- At an initiation meeting, the monitor will comply with the ICH-GCP Guidelines (ICH-GCP 8.3.20) and document that the study procedures were reviewed with the investigator and the investigator's staff.

During the study, a monitor will pay visits to the investigational site in order to:

- provide information and support to the Investigator.
- confirm that facilities remain acceptable.

- confirm that the investigational team is adhering to the CSP.
- confirm that data are being accurately recorded in the CRFs.
- ensure that accountability checks for the IMP are being performed.
- conduct source data verification, which will require direct access to all original records for each subject (e.g. medical records).

The monitor will be available (by phone, and e-mail) between visits whenever the investigator or other study personnel at the investigational site needs information, advice or help.

All documentation and correspondence pertaining to the study (raw data, letters etc.) should be kept in accordance with ICH-GCP.

Audits and inspections

The purpose of an audit or inspection is to systematically and independently examine all study related activities to document that they were conducted, recorded, analyzed and accurately reported according to the CSP and the background regulatory demands.

Audits or inspections may therefore be performed at the study site during or after the study. Visits may thereby be paid by the sponsor or by the MPA. These visits may include source data verification and confidentiality documents are therefore created.

The Investigator should contact the monitor immediately if they are contacted by the MPA about an inspection at their study site.

Training of study personnel

The investigator will maintain records of all individuals involved in the study (medical, nursing and other personnel). The investigator will ensure that appropriate training relevant to the study is given to the personnel involved in the study, and that any new information of relevance to the conduct of the study is forwarded to the persons involved.

Changes to the study protocol

Study procedures will not be changed without mutual agreement between the investigators.

If the clinical study protocol (CSP) needs to be substantially amended, the amendment or a new version of the CSP must be approved by the MPA and the ethical committee before implementation. Approval must also be obtained for the written Patient Information and Informed Consent Form, if applicable.

If an amendment to the CSP substantially alters the study design, or increases the potential risk to the patients, written informed consent must be obtained again for currently enrolled patients and must be provided to additional patients prior to their entry into the study.

If the amendment only involves a new study site, new investigator or new Patient Information, approval must only be approved by the ethical committee.

Protocol deviations

Deviations from the CSP, deemed necessary for an individual subject, will be reported in the CRF giving the reason and date. If necessary, the investigator (or designee) will contact the monitor to inform about the deviation.

Study reporting

After completion of the study, the Investigator will prepare a clinical study report. The Investigator is responsible for submitting the final study report to the MPA.

Archiving

The Investigator shall keep records of the study for 10 years after final signed 12-month Clinical Study Report. This includes any original source documents related to the study, including the subject Identification List with Subject Numbers, full names and the original signed informed consent forms. The sponsor should be contacted before any study related documentation is planned for destruction.

Patient confidentiality

The patients have the right to request access to his/her personal data and the right to request rectification of any data that is not correct and/or complete. Investigator or designee personnel whose responsibilities require access to personal data agree to keep the identity of each patient confidential. This agreement is to be substantiated in a separate document.

Insurance

Participants are covered by "patientskadelagen" and the investigational medicinal product betamethasone is covered by "läkemedelsförsäkringen".

Publication

The investigator is responsible for registering the study in a publicly accessible database before recruitment of the first patient.

The study report may form the basis for a manuscript intended for publication in a medical journal. Attempts to publish negative or inconclusive as well as positive results must be made, or otherwise made publicly available.

Quality control (QC) and quality assurance (QA)

This study will be conducted in compliance with this protocol, standard operating procedures at the study site, the ICH Guideline for GCP and any local regulations.

Audit of the study sites may be conducted to assess and help assure compliance with GCP and applicable regulatory requirements. The study sites may be subject to a QA audit by the sponsor or its representatives, as well as this study may be reviewed by an independent QA department and/or inspected by regulatory authorities. This implies that auditors/inspectors will have the right to inspect the study sites at any time during and/or after completion of the study and will have access to source documents, including patients' medical records. By participating in this study, the investigator agrees to this requirement.

Ethical considerations

The trial has been registered with the EU's electronic database of clinical trials (EudraCT), with EudraCT number 2016-002585-32, and sponsor protocol number mpp01. The trial will be submitted to the Swedish Medical Products Agency (läkemedelsverket) and Regional Ethical Review Board in Stockholm for review. Following approval, the study will be started. Patients will be informed of the study verbally and in writing. Details about the trial including risks and benefits will be explained and any questions answered. A copy of the written consent form will then be signed.
The trial will be conducted in compliance with this protocol, the ICH guidelines for Good Clinical Practice, the latest edition of the Declaration of Helsinki and any local regulations.

Statistics

Power calculation: Was done using www.sealedenvelope.com. The significance level was set at 5% and the power at 90%. In the only randomised trial available, cortisone versus not cortisone resulted in defervescence by 48 hours in 100% and 0% respectively and hypoxemia lasted for a mean <2 versus >3 days, respectively. Our power calculation therefore assumes resolution of hypoxemia by 48 hours in 95% of participants with betamethasone and 65% of those without betamethasone. The sample size required in each group would then be 33. Assuming a 5% loss to follow up 70 patients need to be included.

Duration: Approximately one year for patient inclusion and follow up. The trial will end after the last visit of the last subject. A Declaration of End of Trial Notification will be sent to the MPA at the latest 90 days after the study has ended.

Data storage: All CRFs and signed informed consent forms will be collected from all study sites and stored in a locked cupboard in a locked room at Danderyd hospital for at least 10 years. Data will be double entered using EpiData 3.1 by the investigators at Danderyd Hospital. Study ID only will be used on computerised records. The key will be stored with the CRFs at Danderyd Hospital.

Data analysis: The data will be analysed using survival estimates of the primary endpoint. A per protocol and intention to treat analysis will be done. Loss to follow up will be censored at the time of loss to follow up. Final analysis will include a description of included participants, proportions of adverse events and any serious adverse events, the proportion of participants withdrawn or lost to follow up, the cumulative success and failure rates by week 8. Categorical variables will be compared using the χ^2 test or Fisher exact test and continuous variables using quantile regression.

References

1. Clyde WA, Jr. Clinical overview of typical Mycoplasma pneumoniae infections. Clin Infect Dis. 1993;17 Suppl 1:S32-6.
2. Saraya T, Kurai D, Nakagaki K, Sasaki Y, Niwa S, Tsukagoshi H, et al. Novel aspects on the pathogenesis of Mycoplasma pneumoniae pneumonia and therapeutic implications. Front Microbiol. 2014;5:410.
3. Lee KY, Lee HS, Hong JH, Lee MH, Lee JS, Burgner D, et al. Role of prednisolone treatment in severe Mycoplasma pneumoniae pneumonia in children. Pediatr Pulmonol. 2006;41(3):263-8.
4. Lu A, Wang L, Zhang X, Zhang M. Combined treatment for child refractory Mycoplasma pneumoniae pneumonia with ciprofloxacin and glucocorticoid. Pediatr Pulmonol. 2011;46(11):1093-7.
5. Tamura A, Matsubara K, Tanaka T, Nigami H, Yura K, Fukaya T. Methylprednisolone pulse therapy for refractory Mycoplasma pneumoniae pneumonia in children. J Infect. 2008;57(3):223-8.
6. Luo Z, Luo J, Liu E, Xu X, Liu Y, Zeng F, et al. Effects of prednisolone on refractory mycoplasma pneumoniae pneumonia in children. Pediatr Pulmonol. 2014;49(4):377-80.
7. Huang L, Gao X, Chen M. Early treatment with corticosteroids in patients with Mycoplasma pneumoniae pneumonia: a randomized clinical trial. J Trop Pediatr. 2014;60(5):338-42.
8. Blum CA, Nigro N, Briel M, Schuetz P, Ullmer E, Suter-Widmer I, et al. Adjunct prednisone therapy for patients with community-acquired pneumonia: a multicentre, double-blind, randomised, placebo-controlled trial. Lancet. 2015;385(9977):1511-8.
9. Society BT, Network SIG. British guideline on the management of asthma 2014 [Available from: http://sign.ac.uk/pdf/SIGN141.pdf.
10. El Moussaoui R, Opmeer BC, Bossuyt PM, Speelman P, de Borgie CA, Prins JM. Development and validation of a short questionnaire in community acquired pneumonia. Thorax. 2004;59(7):591-5.
11. Programme CTE. Common Terminology Criteria for Adverse Events version 4.0: National Cancer Institute; 2009 [2016-02-01]. Available from: http://evs.nci.nih.gov/ftp1/CTCAE/CTCAE_4.03_2010-06-14_QuickReference_8.5x11.pdf.
